# Impact of Aortic Root Abscess on Surgical Outcomes of Infective Endocarditis

**DOI:** 10.3390/life14010092

**Published:** 2024-01-07

**Authors:** Ahmed Elderia, Anna-Maria Wallau, Walid Bennour, Stephen Gerfer, Christopher Gaisendrees, Ihor Krasivskyi, Ilija Djordjevic, Thorsten Wahlers, Carolyn Weber

**Affiliations:** Department of Cardiothoracic Surgery, Heart Center, University of Cologne, 50937 Köln, Germanycarolyn.weber@uk-koeln.de (C.W.)

**Keywords:** infective endocarditis, aortic root abscess, paravalvular abscess, aortic valve replacement, aortic root reconstruction, Bentall procedure

## Abstract

Locally destructive infective endocarditis (IE) of the aortic valve complicated by abscess formation in the aortic root may seriously affect patients’ outcomes. Surgical repair of such conditions is often challenging. This is a single-center observational analysis of consecutive patients treated surgically for IE between 2009 and 2019. We divided the cohort into two groups considering the presence of an aortic root abscess and compared the characteristics and postoperative outcomes of patients accordingly. Moreover, we examined three different procedures performed in abscess patients regarding operative data and postoperative results: an isolated surgical aortic valve replacement (AVR), AVR with patch reconstruction of the aortic root (AVR + RR) or the Bentall procedure. The whole cohort comprised 665 patients, including 140 (21.0%) patients with an aortic root abscess and 525 (78.9%) as the control group. The abscess group of patients received either AVR (66.4%), AVR + RR (17.8%), or the Bentall procedure (15.7%). The mean age in the whole cohort was 62.1 ± 14.8. The mean EuroSCORE II was 8.0 ± 3.5 in the abscess group and 8.4 ± 3.7 in the control group (*p* = 0.259). The 30-day and 1-year mortality rates were 19.6% vs. 11.3% (*p* = 0.009) and 40.1% vs. 29.6% (*p* = 0.016) in the abscess compared to the control group. The multivariable regression analysis did not reveal aortic root abscess as an independent predictor of mortality. Rather, age > 60 correlated with 30-day mortality and infection with *Streptococcus* spp. correlated with 1-year mortality. In the analysis according to the performed procedures, KM estimates exhibited comparable long-term survival (log-rank *p* = 0.325). IE recurrence was noticed in 12.3% of patients after AVR, 26.7% after AVR + RR and none after Bentall (*p* = 0.069). We concluded that patients with an aortic root abscess suffer worse short and long-term outcomes compared to other IE patients. The post-procedural survival among ARA patients did not significantly vary based on the procedures performed.

## 1. Introduction

Infective endocarditis (IE) is a serious disease associated with a wide range of complications and steadily rising incidence [1,2]. Approximately one quarter of patients diagnosed with IE will go on to develop an aortic root abscess (ARA) [3]. Locally destructive IE of the aortic valve complicated by an abscess formation in the aortic root could severely affect patients’ outcomes. Therefore, the diagnosis of an ARA indicates urgent surgery within 3–5 days [1]. Surgical repair of ARA is often challenging. It comprises thorough debridement of the infected tissue followed by annular and, if needed, root reconstruction as well as valve replacement [3,4,5]. Currently, many approaches are available for the management of ARA, and there are no definite preferences outlined in the guidelines to choose among them [1]. Three main approaches are isolated surgical aortic valve replacement (AVR), AVR with patch reconstruction of the aortic root (AVR + RR) and the Bentall procedure. Each of these approaches has its own unique characteristics in terms of feasibility, potential complications and overall outcomes. The selection between these procedures relies on patient demographics and local findings as well as the preferences of the operating surgeon [5].

The primary objective of our study is to enhance our understanding of the characteristics and predisposing factors that influence the clinical course and the surgical outcomes of patients with ARA. Additionally, we aimed to offer an insight into the outcomes of three distinct surgical approaches for managing ARA patients. To achieve this, we conducted a comprehensive analysis to determine the following:

(i) Differences in clinical presentation, comorbidities and microbiological findings between patient populations categorized by the presence of an ARA.
(ii) Disparities in short and long-term survival rates when compared to a control group.
(iii) Whether ARA itself or other predictive factors were correlated with mortality.

Furthermore, we subdivided the ARA patient population based on the performed surgical procedure to achieve the following aims:

(iv) Explore differences in postoperative results and recurrence rates;
(v) Investigate disparities in short and long-term survival outcomes within this subgroup.

## 2. Materials and Methods

### 2.1. Study Design and Population 

This is a single-center analysis of consecutive patients who underwent surgical treatment for IE between 2009 and 2019. We divided the cohort into two groups according to the presence of an ARA. Preoperative demographics, patient-specific risk factors, microbiological findings as well as postoperative complications, length of hospital and intensive care unit (ICU) stay, short- and long-term mortality rates were evaluated. Additionally, we subdivided the ARA patient population into three groups based on the performed procedures: an isolated surgical aortic valve replacement (AVR) group, AVR with patch reconstruction of the aortic root (AVR + RR) group and Bentall group. This allowed us to perform a more detailed examination of the patients’ conditions, operative data and postoperative results as well as short- and long-term survival accordingly. 

The clinical status, microbiological data and echocardiographic findings for each patient were thoroughly assessed with appropriate evaluation of the operative risk and the optimal timing of the procedure. An interdisciplinary endocarditis team consensually chose the antimicrobial regime and the duration of therapy according to, at the time of surgery, recent guidelines [6]. Surgery was indicated according to the guidelines for the management of infective endocarditis of the European Society of Cardiology (ESC) and European Association for Cardio-Thoracic Surgery (EACTS) [6]. All operations were performed under general anesthesia via median sternotomy, with routine establishment of cardiopulmonary bypass (CPB) techniques utilizing roller head pumps, a membrane oxygenator, cardiotomy suction, moderate systemic hypothermia (34 °C) and cardioplegic arrest. 

Relevant data were extracted out of the patients’ digital records and operation reports. Long-term follow-up was obtained by reviewing hospital medical records and conducting interviews with patients, their relatives or their physicians. The institutional ethics committees (Ethics Committee of the Medical Faculty, University of Cologne, 17-407) approved the study protocol.

### 2.2. Definition of Variables

The diagnosis of IE followed the modified Duke criteria, and indication for surgical management adhered to the current guidelines outlined by the European Society of Cardiology [1,7]. The patients’ age was recorded at the time of surgery for IE. The diagnosis of ARA was established through preoperative echocardiography and/or confirmed intraoperatively. We used EuroSCORE II to estimate the perioperative mortality risk. Patients who underwent the sole replacement of an aortic valve were added to the AVR group, while patients who additionally received a pericardial patch reconstruction of the aortic valve annulus, ascending aorta or the left ventricular outflow tract were categorized into the AVR + RR group. The Bentall procedure consisted of a composite valved conduit, which was inserted in the aortic annulus followed by re-implantation of the coronary arteries. The entire aortic root with the aortic valve and part of the ascending aorta were thus replaced [8]. Known previous cerebrovascular events (CVE) were defined as ischemic or hemorrhagic cerebral insults in relation to IE. Postoperative CVE was considered as any new-onset neurologic deficit of cerebral origin, in association with signs of hemorrhage or ischemia on CT/MRI of the brain along with an assessment by a neurologist that occurred during the primary hospital stay. Acute kidney injury (AKI) was defined according to the Kidney International Supplements of 2012 [9]. In addition, 30-day and 1-year mortality included death from any cause within the first 30 days and between day 31 and day 365 after surgery, respectively. Late mortality was defined as all-cause mortality occurring during the follow-up period. The follow-up time for survival was measured from the date of operation to either the date of death or the date of the last contact with the patient. 

### 2.3. Statistical Analysis

All data were statistically analyzed using SPSS^®^ Statistics version 28.0 (IBM Corporation, Armonk, NY, USA). Depending on the distribution, continuous variables were expressed as mean and standard deviation or median with the respective interquartile range. Group comparisons were performed using the unpaired Student’s *t*-test or Mann–Whitney U-test. Discrete variables were expressed as percentages and tested with the chi-squared test or Fisher’s exact test. Missing data were not imputed and were randomly assumed to be missing. Potential risk factors for 30-day mortality (days 1 to 30) were assessed using logistic regression and potential risk factors for 1-year mortality (days 31 to 365) using Cox regression. After univariable analysis, all variables with a *p* value less than 0.1 were entered into the multivariable model using forward selection (likelihood ratio, *p* less than 0.05). The results are presented as odds ratios (OR) for 30-day mortality or hazard ratios (HR) for 1-year mortality, with a corresponding 95% confidence interval (CI) and *p* value. All the reported *p* values are two-sided and considered statistically significant if they were 5% or less. We also calculated Kaplan–Meier (KM) curves to visualize the cumulative survival in the study groups during the follow-up period.

## 3. Results

### 3.1. Results Based on the Presence of an ARA

Of all the patients with surgically treated IE, 21.1% (*n* = 140/665) presented with an ARA. The mean age of patients in the abscess group was 60.8 ± 15.1 comparable to the whole cohort with 62.1 ± 14.8 (*p* = 0.244). The majority of patients were males with 73.2% and 74.8%, respectively (*p* = 0.706). There were no differences regarding the distribution of comorbidities between the abscess group and the non-abscess group. The mean EuroSCORE II in the abscess group was 8.0 ± 3.5 vs. 8.4 ± 3.7 in the non-abscess group (*p* = 0.259). The majority of patients underwent antimicrobial therapy preoperatively with a mean of 14.8 ± 14.7 days in the abscess group vs. 15.2 ± 14.5 days in the non-abscess group (*p* = 0.848). Baseline data are listed in Table 1. Risk factors of IE are listed in Supplementary Table S1. 

In the abscess group, *Streptococcus* spp. was the most commonly identified microorganism with 33.1% followed by Staphylococcus aureus with 23.7%, whereas *Streptococcus* spp. was with 25.0% slightly less identified than Staphylococcus aureus with 26.7% in the non-abscess group. There were no statistically significant variations between groups. Microbiological data are listed in Table 2. 

In the abscess group, paravalvular perforation was more frequently detected, either in the preoperative echocardiography or intraoperatively, at 24.5%, compared to 15.3% in the non-abscess group (*p* = 0.011). Other clinical, laboratory and echocardiographic manifestations of IE are listed in Supplementary Table S2. One-third of the examined population exhibited an additional mitral valve infection, with no significant divergence in distribution between the groups (*p* = 0.081). In the abscess group, mitral valve procedures were performed less frequently, at 30.2%, compared to 38.7% in the non-abscess group (*p* = 0.064). Prosthetic aortic valve endocarditis was noticed in 20.1% and 16.6% of patients in the abscess and non-abscess groups respectively (*p* = 0.327). There were no significant variations between groups regarding operation, bypass and cross-clamp times. Operative data are listed in Table 3.

**Figure 1:**
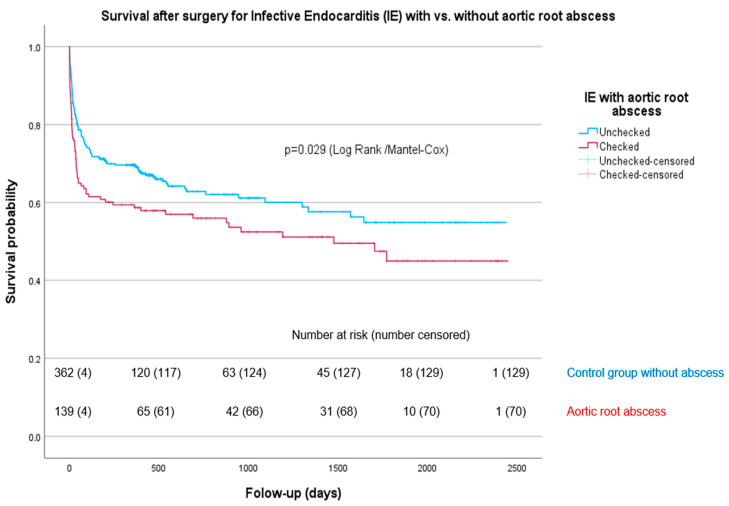
Survival after surgery for IE in patients with vs. without aortic root abscess.

The abscess group had a higher 30-day mortality than the non-abscess group with 19.6% vs. 11.3%; (*p* = 0.009). Likewise, 1-year mortality was significantly higher in the abscess group; (*p* = 0.016). The leading cause of death in both groups was septic shock, accounting for 35.6% of cases, followed by multiple organ failure at 16.3% (see Supplementary Table S3). KM estimates for cumulative survival in both groups are visualized in Figure 1 (log-rank *p* = 0.029).

Significantly more patients in the abscess group underwent new pacemaker implantation, at 14.9%, compared to the non-abscess group at 9.1%; (*p* = 0.044). Moreover, IE recurrence rates were significantly higher in the abscess group, at 12.5%, compared to the non-abscess group at 4.4% (*p* = 0.005). On the other hand, perioperative new CVE and AKI occurred similarly in both groups. The duration of stay in the ICU and in the hospital was similar in both groups. Approximately half of the patients in the studied population were readmitted following their discharge, mainly because of sepsis and/or cardiac decompensation, with no significant differences observed between the groups. Outcomes are listed in Table 4. 

To identify relevant predictors of 30-day and 1-year mortality, we performed a univariable and multivariable regression analysis. In the multivariable analysis, only age > 60 years and infection with Staphylococcus aureus correlated with 30-day mortality. 

For 1-year mortality, our multivariable analysis did not show significant correlation of ARA to 1-year mortality. Rather, infection with *Streptococcus* spp. and LVEF < 30% correlated to 1-year mortality (see Table 5). 

### 3.2. Results According to Performed Procedures in Patients with ARA

The second analysis according to the performed procedures in patients with ARA revealed similar demographics and equal distribution of comorbidities and risk scores (see Table 6). Likewise, operative times were comparable in all three groups. Biological valve prostheses were more frequently used than mechanical valve prostheses with no distribution differences between the three groups. Table 7 shows the operative data of patients with ARA according to the performed procedure.

**Figure 2:**
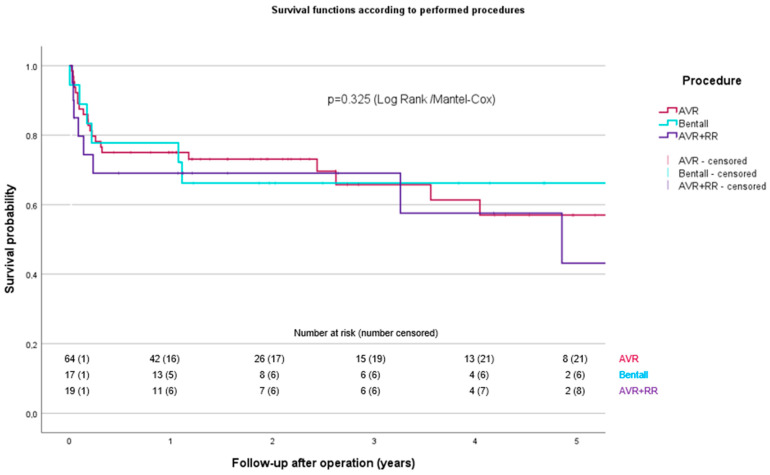
Survival curves using the Kaplan–Meier method for patients with ARA based on the procedures conducted. AVR: aortic valve replacement, AVR + RR: aortic valve replacement + root reconstruction, ARA: aortic root abscess.

There were no statistically significant differences in 30-day and 1-year mortality between the three groups. Survival curves using the KM method for patients with ARA based on the procedures conducted are shown in Figure 2.

**Figure 3:**
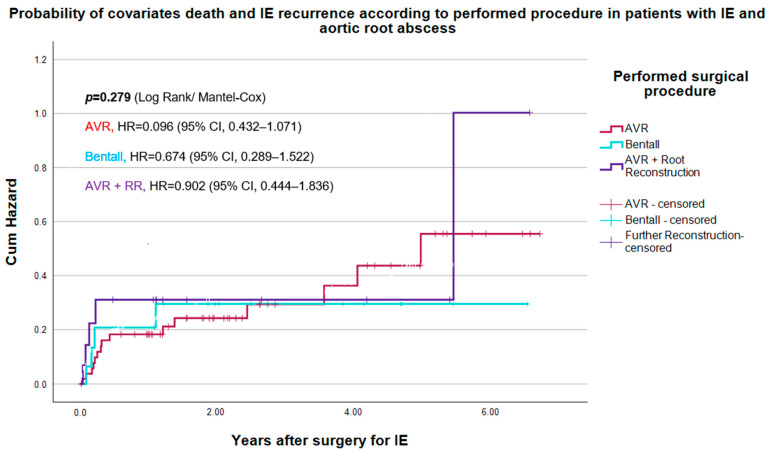
Probability of death and/or recurrence of IE according to performed procedures in patients with IE complicated with aortic root abscess.

No significant variations were found regarding perioperative complications like CVE and new pacemaker implantation, as listed in Table 8. Similar high AKI and readmission rates were noticed after all three procedures. However, IE recurrence was noticed in 12.3% of patients after AVR, 26.7% after AVR + RR; (*p* = 0.069) and none after Bentall (*p* = 0.095). There appeared to be a trend with almost twice as much recurrence after AVR + RR compared to AVR and none after Bentall, even though it was not significant. Figure 3 displays the probability of death and/or IE recurrence in patients with ARA based on the procedures conducted (log-rank *p* = 0.279).

## 4. Discussion

Although IE is a rare disease, it exhibits a rising incidence and outcomes that need to be improved, so there is a continued demand for research in this field [1,2]. In total, 21% of patients who underwent surgery for IE at our institution had an ARA. Our objective in undertaking this work was to contribute to a deeper comprehension of this condition and provide insights for better treatment of individuals at risk. Furthermore, we compared the outcomes of three common surgical techniques for managing ARA, aiming at providing guidance in borderline cases. Our findings suggested that despite presentation with similar baseline characteristics, ARA patients suffered significantly higher short- and long-term mortality compared to non-abscess patients. Nevertheless, the presence of ARA was not found to be an independent predictor of mortality. There were no differences in postoperative outcomes or short- and long-term survival in the analysis according to the performed procedures in patients with ARA. A tendency towards less IE recurrence was noticed after the Bentall procedure compared to isolated AVR or AVR + RR. However, the hazards for the covariates death and/or recurrence were in turn comparable between the three groups.

ARA as a complication of locally destructive IE is a dynamic process that typically begins with wall thickening in the aortic root, which commonly extends into the aortomitral intervalvular fibrosa and can progress further, potentially leading to a fistula [10,11]. This comprises an obstacle in controlling infection, which indicates surgical eradication in addition to antimicrobial therapy. Therefore, the presence of an ARA is considered a case of surgical urgency according to the ESC/EACTS guidelines 2023 [1]. Remarkably, in the updated version, as compared to the 2015 guidelines [12], that definition of surgical urgency, which was previously indicated ‘’as soon as possible’’, is now quantified as within 3–5 days. In the examined population here, ARA patients awaited a mean of 14.5 days from the diagnosis until surgical intervention. This might have negatively affected the outcomes.

As previously described by Harris et al., the presence of an ARA was not found to be an independent risk factor of mortality in our multivariable regression analysis [13]. Rather, age over 60 years and IE caused by Staphylococcus aureus were identified as independent predictors for 30-day mortality, whereas infection with *Streptococcus* spp. and LVEF < 30% correlated with 1-year mortality. Both advanced age and infection with Staphylococcus aureus are evidenced predictors of worse survival in surgically treated IE patients [14,15,16]. Higher ejection fraction was found to be associated with better survival after surgery for PVE [17]. Similar to our results, Rouzé et al. reported that *Streptococcus* spp. infection was associated with severe local destruction, a higher incidence of paravalvular complications and was identified as an independent predictor of reoperation and worse long-term survival [18].

In our analysis, *Streptococcus* spp. followed by Staphylococcus aureus and thirdly CONS were the most commonly identified microorganisms. Other studies showed that CONS is the most commonly identified microorganism in patients with ARA [19,20]. On the other hand, *Streptococcus* spp. was identified as the most aggressive microorganism with severe paravalvular complications, including abscess or fistula [18].

The local destruction of the aortic valve ring anatomically raises the risk of atrioventricular block (AVB) and pacemaker dependency, as observed in our study. It is also possible that the massive destruction of the valve and/or root tissue predispose patients to septic embolization, yet the rates of CVE were comparable between groups. The presence of paravalvular fistula was identified in 4.3% of ARA patients. This observation could potentially account for the high recurrence rate of 12.5% in this group. It is evidenced that a radical debridement of infected tissue is essential as a prerequisite for achieving optimal infection control and improving the prognosis. Distinguishing between cases of recurrence caused by relapse or reinfection is a matter that warrants further research and investigation.

For the optimal eradication of infection and reconstruction of the aortic root in ARA patients, the current literature does not support a standard surgical approach [1]. Isolated AVR counts as a routine and safe procedure, while the Bentall operation carries a higher risk of complications. In the examined population, there was no observed increase in the incidence of postoperative complications in the Bentall group, suggesting that a careful technique in the reimplantation of the coronary arteries and aortic root can effectively mitigate the risk of postoperative myocardial ischemia. Divergent from our results, another study found that the Bentall procedure was associated with a longer operation time, longer bypass time and aortic clamping time, which has been shown to increase in-hospital mortality [21]. Although the AVR + RR group had a lower mean EuroSCORE II compared to the other groups, the mortality rate within the initial 30 days was the highest in this group in terms of percentages. However, this difference did not reach statistical significance. Moreover, almost half of the patients in the AVR + RR group developed AKI perioperatively, half of whom required dialysis. This contradicts the outcome of a retrospective study from Innsbruck [22] in which aortic root reconstruction was associated with a shorter operation time, less need for circulatory support perioperatively and better short- and long-term survival rates, when compared to root replacement.

It is worth noting that whether the abscess cavity was directly closed with a suture during isolated AVR, sealed using a bovine pericardial patch or managed through complete excision of the aortic root, all of these approaches resulted in similar 30-day and 1-year mortality rates. Here, it is essential to consider a thorough debridement of the infected tissue, which outweighs the operative risk associated with performing more complex procedures. Our findings appear to support this, as none of the patients in the Bentall group experienced IE recurrence during the follow-up period. On the other hand, the group that underwent AVR + RR exhibited a recurrence at 26%, although this difference was not statistically significant. Presumably, AVR was performed in less severe cases, while AVR + RR and Bentall were preferred in more advanced cases. It is possible that the greater extent of tissue debridement achieved through Bentall procedures contributed to the lower recurrence rate observed in this group.

Similar to our results, Harris et al. reported comparable survival with less reoperation rates after root replacement compared to root reconstruction [13]. In a meta-analysis conducted by Chen et al., it was observed that patients who underwent root replacement exhibited similar short- and long-term mortality rates when compared to those who underwent root reconstruction. However, the recurrence rate favored the group that received root replacement, implying that better infection eradication was achieved through this approach [23]. In their study of 148 patients with ARA, Gollmann-Tepeköylü et al. found that the 5-year survival rate following annular reconstruction using bovine pericardium + AVR was approximately 75%, which is comparable to our result with 70%. Additionally, they reported that after a median follow-up of 9 years, patients who underwent root repair + AVR had better event-free survival rates and higher instances of IE recurrence, but comparable reoperation rates when compared to those who received root replacement with a freestyle xenograft prosthesis [22]. However, the authors suggested that the decision of which surgical approach to pursue may have been influenced by the extent of root destruction, with replacement having been more common in patients with more advanced disease, potentially impacting the outcomes. The use of homografts in patients with ARA was previously examined, exhibiting lower rates of IE recurrence, which makes it a good alternative especially in cases of PVE. However, its restricted availability and concerns about structural valve degeneration and higher risks of reoperation limit its use [18,22,24]. In the study period, only a few patients received homografts due to ARA in our institution and were hence excluded from this analysis. In their study of 168 patients with ARA investigation into AVR with biological or mechanical valves, with or without patch reconstruction of the aortic root, stentless valve, aortic allograft, or composite valve graft, Elgalad et al. found that the outcomes remained unaffected by the surgical complexity of the aortic reconstruction approach or the choice of valve type [25]. They concluded, as we do, that the choice of the most suitable surgical procedure should be customized to match the specific characteristics and needs of each individual patient.

### Limitations

Our analysis has surely some inherent limitations. The presented data are related to a group of patients from a single center. Although we present a relatively large collective of surgically treated IE patients, compared to the available literature, the power of the analysis could have been increased by a larger number of patients as well as by a multicenter study. A higher completeness of follow-ups would have strengthened our statement. The subdivision according to surgical procedures resulted in small groups and a larger or multicenter approach would increase the statistical power. Unfortunately, anatomical specific data, which favored one of the procedures, are not available. Finally, distinguishing between cases of recurrence caused by relapse or reinfection is a matter that warrants further research and investigation.

## 5. Conclusions

Despite having comparable baseline characteristics, patients with ARA exhibited worse short and long-term outcomes compared to other IE patients. The post-procedural outcomes among ARA patients did not significantly vary based on the procedures performed. There appeared to be a tendency towards less IE recurrence following the Bentall procedure compared to isolated AVR or AVR + RR. However, the hazards for the covariates death and/or recurrence were comparable across the three groups. Patient individualized decision making and further studies are warranted.

## Figures and Tables

**Table 1:** Demographics and comorbidities of patients surgically treated for IE.

Variables	All Patients *n* = 665	Non-Abscess Group *n* = 524	Abscess Group *n* = 140	*p*-Value
Age (years)	62.1 ± 14.8	62.5 ± 14.7	60.8 ± 15.1	0.244
Female Gender	169/663 (25.5%)	132 (25.2%)	37 (26.8%)	0.706
BMI (kg/m^2^)	27.3 ± 12.7	26.8 ± 5.7	28.9 ± 25.5	0.373
LVEF < 30%	17/634 (2.7%)	14/500 (2.8%)	3/134 (2.2%)	0.925
LVEF 30–50%	152/634 (24.0%)	119/500 (23.8%)	33/134 (24.6%)	
LVEF > 50%	465/634 (73.3%)	367/500 (73.4%)	98/134 (73.1%)	
HTN	418/665 (62.9%)	339/524 (64.7%)	79/139 (56.8%)	0.088
PHT	51/665 (7.7%)	40/524 (7.6%)	11/139 (7.9%)	0.912
Hyperlipidemia	189/665 (28.4%)	151/524 (28.8%)	38/139 (27.3%)	0.731
CAD	194/665 (29.2%)	148/524 (28.2%)	46/139 (33.1%)	0.264
PVD	62/665 (9.3%)	52/524 (9.9%)	10/139 (7.2%)	0.326
Diabetes mellitus	167/665 (25.1%)	133/524 (25.4%)	34/139 (24.5%)	0.824
Known CVE	86/665 (12.9%)	68/524 (13.0%)	18/139 (12.9%)	0.993
Active smoker	149/665 (22.4%)	123/524 (23.5%)	26/139 (18.7%)	0.231
COPD	72/665 (10.8%)	58/524 (11.1%)	14/139 (10.1%)	0.737
CKD	316/663 (47.7%)	245/524 (46.8%)	71/139 (51.1%)	0.364
Previous cardiac surgery	185/663 (27.9%)	149/524 (28.4%)	36/139 (25.9%)	0.553
Previous valve replacement	154/665 (23.2%)	123/524 (23.5%)	31/139 (22.3%)	0.771
EuroSCORE II (%)	8.3 ± 3.7	8.4 ± 3.7	8.0 ± 3.5	0.259
Preoperative antibiotics	614/656 (93.6%)	483/518 (93.2%)	131/138 (94.9%)	0.473
Duration of antibiotics preoperatively (days)	15.0 ± 14.6	15.2 ± 14.5	14.8 ± 14.7	0.848
Time between diagnosis and operation (days)	17.0 ± 44.6	17.1 ± 47.5	14.5 ± 20.2	0.546

Metric variables are calculated as mean with respective standard deviation (±). For nominal variables, the absolute number (*n*) is calculated with percentages (%). IE: infective endocarditis, BMI: body mass index, LVEF: left ventricular ejection fraction, HTN: arterial hypertension, PHT: pulmonary hypertension, CAD: coronary artery disease, PVD: peripheral vascular disease, CVE: cerebrovascular events, COPD: chronic obstructive pulmonary disease; CKD: chronic kidney disease.

**Table 2:** Microorganisms identified in patients with IE treated surgically.

Variables	All Patients *n* = 665	Non-Abscess Group *n* = 525	Abscess Group *n* = 140	*p*-Value
Known microorganism	597/658 (90.7%)	466/520 (89.6%)	131/138 (94.9%)	0.056
Positive blood culture	537/598 (89.8%)	419/465 (90.1%)	116/131 (88.5%)	0.603
Negative blood culture	61/598 (10.2%)	46/465 (9.9%)	15/131 (11.5%)	
Positive tissue culture	210/590 (35.6%)	170/458 (37.1%)	39/130 (30.0%)	0.135
Negative tissue culture	380/590 (64.4%)	288/458 (62.9%)	91/130 (70%)	
Staphylococcus aureus	173/665 (26.0%)	140/524 (26.7%)	33/139 (23.7%)	0.477
CoNS	92/665 (13.8%)	69/524 (13.2%)	23/139 (16.5%)	0.306
*Streptococcus*	178/665 (26.8%)	131/524 (25.0%)	46/139 (33.1%)	0.055
Gram-negative HACEK	5/665 (0.8%)	4/524 (0.8%)	1/139 (0.7%)	0.958
Gram-negative non-HACEK	25/665 (3.8%)	21/524 (4.0%)	4/139 (2.9%)	0.534
Fungi	9/665 (1.4%)	7/524 (1.3%)	2/139 (1.4%)	0.926
Other organisms	51/665 (7.7%)	42/524 (8.0%)	9/139 (6.5%)	0.545

For listed nominal variables, the absolute number, *n*, is calculated with percentages (%). IE: infective endocarditis. CoNS: coagulase-negative staphylococci. HACEK: *Haemophilus* species, *Aggregatibacter* species, *Cardiobacterium hominis*, *Eikenella corrodens* and *Kingella* species.

**Table 3:** Operative data of patients surgically treated for IE.

Variables	All Patients *n* = 665	Non-Abscess Group *n* = 525	Abscess Group *n* = 140	*p*-Value
Aortic valve procedure	446/663 (67.3%)	306/524 (58.4%)	140/140 (100%)	0.051
Mitral valve procedure	246/665 (37.0%)	203/524 (38.7%)	42/139 (30.2%)	0.064
Tricuspid valve procedure	43/663 (6.5%)	35/524 (6.7%)	8/139 (5.8%)	0.694
Pulmonary valve procedure	3/663 (0.5%)	0/524	3/139 (2.2%)	<0.001
PVE of the aortic valve	115/665 (17.3%)	87/524 (16.6%)	28/139 (20.1%)	0.327
PVE of the mitral valve	36/665 (5.4%)	32/524 (6.1%)	4/139 (2.9%)	0.135
Combined procedure	256/650 (39.4%)	192/513 (37.4%)	64/137 (46.7%)	0.059
*Simultaneous CABG*	78/663 (11.8%)	57/524 (10.9%)	21/139 (15.1%)	0.169
*Simultaneous aortic procedure*	23/665 (3.5%)	18/524 (3.4%)	5/139 (3.6%)	0.926
*Simultaneous ASD-closure*	20/665 (3.0%)	17/524 (3.2%)	3/139 (2.2%)	0.506
*Simultaneous VSD-closure*	9/665 (1.4%)	6/524 (1.1%)	3/139 (2.2%)	0.359
Operation time in minutes	216.3 ± 78.4	210.8 ± 75.3	217 ± 79.2	0.345
CPB time in minutes	126.9 ± 58.7	123.9 ± 59.9	127.9 ± 58.4	0.481
Cross-clamp time in minutes	79.8 ± 35.3	78.0 ± 35.0	80.4 ± 35.5	0.481
Mechanical circulatory support				
ECMO	4/644 (0.6%)	4/525 (0.8%)	0/140 (0%)	0.294
IABP	13/644 (2.0%)	13/525 (2.5%)	0/140 (0%)	
ECMO + IABP	4/644 (0.6%)	3/525 (0.6%)	1/140 (0.7%)	

Metric variables are calculated as mean with respective standard deviation (±). For nominal variables, the absolute number (*n*) is calculated with percentages (%). IE: infective endocarditis, CABG: coronary artery bypass graft operation, ASD: atrial septal defect, VSD: ventricular septal defect, PVE: prosthetic valve endocarditis. CPB: cardiopulmonary bypass, ECMO: extracorporeal membrane oxygenation, IABP: intra-aortic balloon pump.

**Table 4:** Outcomes of surgically treated IE patients with vs. without aortic root abscess.

Variables	All Patients *n* = 665	Non-Abscess Group *n* = 525	Abscess Group *n* = 140	*p*-Value
30-day mortality	82/659 (12.4%)	55/487 (11.3%)	27/138 (19.6%)	**0.009**
1-year mortality	164/659 (24.9%)	108/365 (29.6%)	56/138 (40.1%)	**0.016**
New pacemaker implantation	64/659 (9.7%)	44/485 (9.1%)	20/134 (14.9%)	**0.044**
Re-thoracotomy	99/658 (15.0%)	85/525 (16.2%)	14/140 (10.0%)	0.134
Tracheotomy	90/658 (13.7%)	72/520 (13.8%)	18/136 (13.2%)	0.854
Myocardial infarction	6/656 (0.9%)	5/518 (1.0%)	1/138 (0.7%)	0.792
New CVE	32/656 (4.9%)	26/518 (5.0%)	6/138 (4.3%)	0.814
*TIA*	2/32 (6.3%)	1/26 (3.8%)	1/6 (16.7%)	0.292
*Ischemia*	25/32 (78.1%)	20/26 (76.9%)	5/6 (83.3%)	
*Hemorrhage*	5/32 (15.6%)	5/26 (19.2%)	0/6	
AKI	208/659 (31.6%)	168/519 (32.4%)	40/138 (29.0%)	0.447
*Dialysis*	91/205 (44.4%)	74/165 (44.8%)	17/40 (42.5%)	0.103
Intubation time in hours	171.3 ± 589.9	155.6 ±369.5	176.8 ± 643.4	0.806
ICU stay in days	7.6 ± 8.7	7.0 ± 6.9	7.8 ± 9.1	0.309
In-hospital stay in days	16.6 ± 20.7	14.9 ± 9.3	17.1 ± 23.0	0.291
Readmission during follow up	210/410 (51.2%)	158/320 (49.4%)	51/89 (57.3%)	0.186
*Due to sepsis*	37/210 (17.6%)	27/350 (7.7%)	10/97 (10.3%)	0.412
*Wound infection*	20/210 (9.5%)	17/350 (4.9%)	2/97 (2.1%)	0.227
*New CVE*	25/447 (5.6%)	18/350 (5.1%)	7/97 (7.2%)	0.432
*Cardiac decompensation*	29/210 (13.8%)	19/350 (5.4%)	10/97 (10.3%)	0.084
*Respiratory failure*	17/210 (8.0%)	11/350 (3.1%)	6/97 (6.2%)	0.166
*Other reasons*	24/447 (5.4%)	21/350 (6.0%)	3/97 (3.1%)	0.261
IE recurrence	25/405 (6.2%)	14/317 (4.4%)	11/88 (12.5%)	**0.005**
*Aortic valve involvement*	17/447 (3.8%)	11/350 (3.1%)	6/97 (6.2%)	0.166
*Operative Therapy*	14/20 (70.0%)	10/12 (83.3%)	4/8 (50%)	0.111
*Conservative Therapy*	6/20 (30.0%)	2/12 (16.7%)	4/8 (50%)	

Metric variables are calculated as mean with respective standard deviation (±). For nominal variables, the absolute number (*n*) is calculated with percentages (%). IE: infective endocarditis, ICU: intensive care unit, CVE: cerebrovascular events, TIA: transient ischemic attack, AKI: acute kidney injury. **Bold** indicates *p* < 0.05.

**Table 5:** Independent predictors of mortality among surgically treated IE patients with aortic root abscess.

	Odds/Hazard Ratio [95% Confidence Interval]	*p*-Value
**30-day mortality**		
Age > 60 years	OR 7.917 [0.944–66.419]	<0.001
Staphylococcus aureus infection	OR 3.638 [0.946–13.998]	0.057
**1-year mortality**		
*Streptococcus* infection	HR 1.747 [1.106–2.761]	0.015
LVEF < 30%	HR 1.578 [1.102–2.259]	0.001

IE: infective endocarditis, LVEF: left ventricular ejection fraction.

**Table 6:** Characteristics of patients with aortic root abscess according to the performed procedure.

Variables	AVR Group *n* = 93	*p*-Value	AVR + RR Group *n* = 25	*p*-Value	Bentall Group *n* = 22	*p*-Value
Age < 60 years	43/92 (46.7%)	0.067	9/24 (37.5%)	0.677	5/17 (22.7%)	0.054
Age > 60 years	49/92 (53.3%)		15/24 (62.5%)		17/22 (77.3%)	
Female gender	26/92 (28.3%)	0.587	7/24 (29.2%)	0.774	5/22 (22.7%)	0.637
LVEF < 30%	1/89 (1.1%)	0.470	1/23 (4.3%)	0.723	0/22	0.717
LVEF 30–50%	22/89 (24.7%)		5/23 (21.7%)		6/22 (27.3%)	
LVEF > 50%	66/89 (74.2%)		17/23 (73.9%)		16/22 (72.7%)	
IE history	7/92 (7.6%)	0.447	0/25		2/22 (9.1%)	0.587
HTN	48/92 (52.2%)	0.121	15/25 (60%)	0.724	16/22 (72.7%)	0.101
PHT	10/92 (10.9%)	0.071	1/25 (4.0%)	0.423	0/22	
CAD	29/92 (31.5%)	0.582	9/25 (36.0%)	0.733	7/22 (31.8%)	0.890
DM	22/92 (23.9%)	0.834	7/25 (28.0%)	0.649	5/22 (22.7%)	0.837
Known CVE	11/92 (12.0%)	0.626	3/25 (12.0%)	0.876	4/22 (18.2%)	0.426
CKD	46/92 (50%)	0.722	13/25 (52.0%)	0.919	12/22 (54.5%)	0.723
Active smoker	18/92 (19.6%)	0.716	4/25 (16.0%)	0.702	4/22 (18.2%)	0.945
EuroSCORE II	10.1 ± 3.5	0.577	8.5 ± 3.0	0.329	10.2 ± 2.7	0.222

The *p* values refer to the subanalysis within the abscess group according to the performed procedures, i.e., AVR vs. AVR + RR + Bentall, etc. Metric variables are calculated as mean with respective standard deviation (±). For nominal variables, the absolute number (*n*) is calculated with percentage (%). LVEF: Left ventricular ejection fraction, HTN: arterial hypertension, PHT: pulmonary hypertension, CAD: coronary artery disease, DM: diabetes mellitus, CVE: cerebrovascular events, CKD: chronic kidney disease.

**Table 7:** Operative data of patients with aortic root abscess.

Variables	AVR Group *n* = 93	*p*-Value	AVR + RR Group *n* = 25	*p*-Value	Bentall Group *n* = 22	*p*-Value
Operation time in minutes	192.3 ± 75.3	0.097	222.17 ± 77.2	0.575	213.0 ± 78.5	0.685
CPB time in minutes	112.8 ± 38.5	0.146	126.5 ± 55.4	0.285	125.7 ± 65.8	0.921
Cross-clamp time in minutes	70.3 ± 21.4	0.205	76.8 ± 34.9	0.147	79.9 ± 37.9	0.723
Biological valve implanted	57/93 (61.3%)	0.617	14/25 (56.0%)	0.744	12/22 (54.5%)	0.726
Mechanical valve implanted	35/93 (37.6%)		11/25 (44.0%)		10/22 (45.5%)	
Simultaneous other cardiac procedure	45/91 (49.5%)	0.367	9/24 (37.5%)	0.319	10/22 (45.5%)	0.897
Simultaneous CABG	15/92 (16.3%)	0.582	3/25 (12.0%)	0.632	3/22 (13.6%)	0.834

Metric variables are calculated as mean with respective standard deviation (±). For nominal variables, the absolute number (*n*) is calculated with percentages (%). IE: infective endocarditis, CABG: coronary artery bypass graft operation, CPB: cardiopulmonary bypass.

**Table 8:** Outcomes of patients with aortic root abscess according to the performed procedure.

Variables	AVR Group *n* = 93	*p*-Value	AVR + RR Group *n* = 25	*p*-Value	Bentall Group *n* = 22	*p*-Value
30-day mortality	7/66 (10.6%)	0.730	3/20 (15.0%)	0.577	1/19 (5.3%)	0.351
1-year mortality	16/66 (24.2%)	0.653	6/20 (30%)	0.626	4/19 (21.1%)	0.607
Re-thoracotomy	8/93 (8.6%)	0.332	4/25 (16.0%)	0.407	2/22 (9.1%)	0.731
Myocardial infarction	1/92 (1.1%)	0.478	0/24	0.645	0/22	
New pacemaker implantation	10/92 (10.9%)	0.966	3/24 (12.5%)	0.789	2/21 (9.5%)	0.820
New CVE	4/92 (4.3%)	1.0	1/24 (4.2%)	0.962	1/22 (4.5%)	0.960
AKI	22/92 (23.9%)	0.063	11/24 (45.8%)	0.045	7/22 (31.8%)	0.749
*Postoperative dialysis*	11/22 (50%)	0.304	5/11 (45.5%)	0.801	1/7 (14.3%)	0.233
IE recurrence	7/57 (12.3%)	0.933	4/15 (26.7%)	0.069	0/16 (0.0%)	0.095
Hospital readmission	32/58 (55.2%)	0.578	9/16 (56.3%)	0.925	11/16 (68.8%)	0.307

For nominal variables, the absolute number, *n*, is calculated with percentage (%). AKI: acute kidney injury, CVE: cerebrovascular events, IE: infective endocarditis.

## Data Availability

The data that support the findings of this study are available from the corresponding author upon reasonable request.

## Supplementary Materials

The following supporting information can be downloaded at: https://www.mdpi.com/article/10.3390/life14010092/s1, Supplementary Table S1: Risk factors for infective endocarditis (IE). Supplementary Table S2: Manifestations of infective endocarditis (IE). Supplementary Table S3: Causes of death in surgically treated IE patients.

## Abbreviations

AKI	Acute Kidney Injury
ARA	Aortic Root Abscess
AVB	Atrioventricular Block
AVR	Aortic Valve Replacement
AVR + RR	Aortic Valve Replacement + Root Reconstruction
CI	Confidence Interval
CKD	Chronic Kidney Disease
CPB	Cardiopulmonary Bypass
CVE	Cerebrovascular Events
EuroSCORE II	European System for Cardiac Operative Risk Evaluation II
HR	Hazard Ratio
IE	Infective Endocarditis
IQR	Interquartile Range
OR	Odds Ratio
PVE	Prosthetic Valve Endocarditis
