# Supplementary material for: Impact of Aortic Root Abscess on Surgical Outcomes of Infective Endocarditis
Source: Life (Basel). 2024 Jan 7;14(1):92. doi: 10.3390/life14010092 (PMC10820780; doi:10.3390/life14010092)
Supplement: Supplementary file 1 [file life-14-00092-s001.zip › life-2763244-supplementary.pdf]

Supplementary Table S1: Risk factors for Infective Endocarditis (IE).

**Variables**	**All patients** n = 665	**Non-Abscess group** n = 525	**Abscess group** n = 140	***p*-value**
History of IE	45/665 (6.7%)	36/524 (6.9%)	9/139 (6.5%)	0.869
Valvular heart disease	203/665 (30.5%)	162/524 (30.9%)	41/139 (29.5%)	0.747
Mitral valve prolapse	31/665 (4.7%)	26/524 (5.0%)	5/139 (3.6%)	0.498
Previous cardiac surgery	185/663 (27.9%)	149/524 (28.4%)	36/139 (25.9%)	0.553
Previous valve replacement	154/665 (23.2%)	123/524 (23.5%)	31/139 (22.3%)	0.771
Vascular graft implantation	18/665 (2.7%)	15/524 (2.9%)	3/139 (2.2%)	0.650
Congenital heart disease	19/665 (2.9%)	15/524 (2.9%)	4/139 (2.9%)	0.992
HIV Infection	57/665 (8.6%)	12/524 (2.3%)	3/139 (2.2%)	0.926
Active Malignancy	57/665 (8.6 %)	46/524 (8.8%)	11/139 (7.9%)	0.746
ESRD under dialysis	62/312 (19.9%)	53/242 (21.9%)	9/70 (12.9%)	0.234
Current Alcohol abuse	52/665 (7.8%)	40/524 (7.6%)	12/139 (8.6%)	0.697
Current intravenous drug abuse	44/665 (6.6%)	35/524 (6.7%)	9/139 (6.5%)	0.931
Active Hepatitis	44/663 (6.6%)	34/524 (6.5%)	10/139 (7.2%)	0.766
Spondylodiscitis	46/665 (6.9%)	39/524 (7.4%)	6/139 (4.3%)	0.193
Previous Pacemaker Implantation	56/665 (8.6%)	45/516 (8.7%)	11/135 (8.1%)	0.833

For listed nominal variables, the absolute number, n, is calculated with percentage **(%).**

IE: infective endocarditis, HIV: human immunodeficiency virus, ESRD: End stage renal disease.

Supplementary Table S2: Manifestations of Infective Endocarditis (IE).

Variables	All patients<br>n = 665	Non-Abscess group<br>n = 525	Abscess group<br>n = 140	*p*-value
**Clinical manifestations**				
Fever	376/663 (56.7%)	294/524 (56.1%)	82/139 (59.0%)	0.542
Neurological	183/663 (27.6%)	140/524 (26.7%)	43/139 (30.9%)	0.323
Headache/dizziness	11/54 (20.4%)	7/41 (17.1%)	4/13 (30.8%)	0.608
Disturbance in vision	2/54 (3.7%)	2/41 (4.9%)	0/13	
Seizures	3/54 (5.6%)	2/41 (4.9%)	1/13 (7.7%)	
Paresis/Plegia	5/54 (9.3%)	5/41 (12.2%)	0/13	
Encephalitis/meningitis	11/54 (20.4%)	9/41 (22.0%)	2/13 (15.4%)	
Delirium	22/54 (40.7%)	16/41 (39.0%)	6/13 (46.2%)	
TIA	14/179 (7.8%)	12/136(8.8%)	2/43(4.7%)	0.836
Ischemia	98/179 (54.7%)	73/136 (53.7%)	25/43 (58.1%)	
Bleeding	12/179 (6.7%)	9/136 (6.6%)	3/43 (7.0%)	
Others	55/179 (30.7%)	42/136 (30.9%)	13/43 (30.2%)	
Sepsis	262/663 (39.5%)	208/524 (38.8%)	54/139 (38.8%)	0.856
Preoperative catecholamines	86/663 (13.0%)	71/524 (13.5%)	15/139 (10.8%)	0.390
Septic embolism	219/653 (33.5%)	170/517 (32.9%)	49/136 (36.0%)	0.520
Cerebral embolism	155/663 (23.4%)	122/524 (23.3%)	33/139 (23.7%)	0.910
Preoperative Intubation	99/663 (14.9%)	80/524 (15.3%)	19/139 (13.7%)	0.638
Myocardial infarction	16/663 (2.4%)	15/524 (2.9%)	1/139 (0.7%)	0.143
**Laboratory manifestations**				
Bacteraemia	447/663 (67.4%)	354/524 (67.6%)	93/139 (66.9%)	0.884

Leucocytes preoperatively	10.2±6.8	10.1±5.2	10.7±10.8	0.346
Leucocytes 2. POD	12.5±5.7	12.5±5.4	12.5±6.5	0.924
Leucocytes 7. POD	10.9±5.5	10.8±5.4	11.3±5.9	0.406
**Echocardiographic manifestations**				
Perforation	114/663 (17.2%)	80/524 (15.3%)	34/139 (24.5%)	**0.011**
Fistula	15/663 (2.3%)	9/524 (1.7%)	6/139 (4.3%)	0.067
Vegetations	531/650 (81.7%)	423/514 (82.3%)	108/136 (79.4%)	0.439

Metric variables are calculated as mean with respective standard deviation (±). For nominal variables, the absolute number (n) is calculated with percentage (%).

TIA: Transient ischemic attack, POD: Postoperative day.

Supplementary Table S3: Causes of death in surgically treated IE patients

**Variables***	**All patients** (n = 665)
Septic shock	37/104 (35.6%)
Multiorgan failure	17/104 (16.3%)
Cardiogenic shock	12/104 (11.5%)
Right-sided heart failure	1/104 (1.0%)
Myocardial infraction	2/104 (1.9%)
Pericardial tamponade	8/104 (7.7%)
Respiratory failure	8/104 (7.7%)
Mesenterial ischemia	3/104 (2.9%)
Ischemic stroke	2/104 (1.9%)
Intracranial haemorrhage	3/104 (2.9%)
Perioperative bleeding complications	5/104 (4.8%)
Major bleeding	3/104 (2.9%)
Malignancy	4/104 (3.8%)
IE recurrence	5/104 (4.8%)
ARF	1/104 (1.0%)
Others	9/104 (8.7%)

*For listed nominal variables, the absolute number, n, is calculated with percentage (%).

IE: infective endocarditis, ARF: acute renal failure.
